# Screening for obstructive sleep apnea in patients with cancer — a machine learning approach

**DOI:** 10.1093/sleepadvances/zpad042

**Published:** 2023-10-31

**Authors:** Karen A Wong, Ankita Paul, Paige Fuentes, Diane C Lim, Anup Das, Miranda Tan

**Affiliations:** Pulmonary Service, Department of Medicine, Memorial Sloan Kettering Cancer Center, New York, NY, USA; Department of Electrical and Computer Engineering, Drexel University, Philadelphia, PA, USA; Department of Medicine, Memorial Sloan Kettering Cancer Center, New York, NY, USA; Department of Medicine, Miami Veterans Affairs Healthcare System, Miami, FL, USA; Department of Medicine, University of Miami, Miami, FL, USA; Department of Electrical and Computer Engineering, Drexel University, Philadelphia, PA, USA; Pulmonary Service, Department of Medicine, Memorial Sloan Kettering Cancer Center, New York, NY, USA

**Keywords:** machine learning, cancer, OSA-clinical assessment

## Abstract

**Background:**

Obstructive sleep apnea (OSA) is a highly prevalent sleep disorder associated with daytime sleepiness, fatigue, and increased all-cause mortality risk in patients with cancer. Existing screening tools for OSA do not account for the interaction of cancer-related features that may increase OSA risk.

**Study Design and Methods:**

This is a retrospective study of patients with cancer at a single tertiary cancer institution who underwent a home sleep apnea test (HSAT) to evaluate for OSA. Unsupervised machine learning (ML) was used to reduce the dimensions and extract significant features associated with OSA. ML classifiers were applied to principal components and model hyperparameters were optimized using k-fold cross-validation. Training models for OSA were subsequently tested and compared with the STOP-Bang questionnaire on a prospective unseen test set of patients who underwent an HSAT.

**Results:**

From a training dataset of 249 patients, kernel principal component analysis (PCA) extracted eight components through dimension reduction to explain the maximum variance with OSA at 98%. Predictors of OSA were smoking, asthma, chronic kidney disease, STOP-Bang score, race, diabetes, radiation to head/neck/thorax (RT-HNT), type of cancer, and cancer metastases. Of the ML models, PCA + RF had the highest sensitivity (96.8%), specificity (92.3%), negative predictive value (92%), F1 score (0.93), and ROC-AUC score (0.88). The PCA + RF screening algorithm also performed better than the STOP-Bang questionnaire alone when tested on a prospective unseen test set.

**Conclusions:**

The PCA + RF ML model had the highest accuracy in screening for OSA in patients with cancer. History of RT-HNT, cancer metastases, and type of cancer were identified as cancer-related risk factors for OSA.

**Statement of Significance**

Obstructive sleep apnea (OSA) is a common sleep disorder that may adversely affect patients with cancer. Current screening tools for OSA have not been validated in patients with cancer and do not include features that may increase the risk for OSA in this population. Our study developed an accurate machine learning model to screen for OSA and identified cancer-related characteristics that may increase the risk for OSA. This model could be used to effectively screen for OSA in patients with cancer and improve access to care.

## Introduction

Obstructive sleep apnea (OSA) is a highly prevalent medical condition with nearly one billion people affected globally [1]. In spite of its rising prevalence [2, 3], up to 80% of patients with moderate-to-severe OSA remain undiagnosed [4]. OSA confers deleterious quality-of-life symptoms (e.g. daytime sleepiness and fatigue) [5, 6] and is associated with numerous medical conditions [7, 8], including cancer [9, 10]. OSA is implicated in oncogenesis [11, 12], tumor progression [13], and all-cause cancer mortality [14–16]. Preclinical [17, 18] and epidemiological studies [14] have proposed that chronic intermittent hypoxia (IH), a pathophysiologic feature of OSA, contributes to tumor growth and metastasis. To date, clinical studies have been done to assess the prevalence of OSA in specific types of cancers [19, 20], yet there has not been an established screening tool for OSA in patients with cancer.

OSA is characterized by recurrent upper airway (UA) collapse during sleep, resulting in reduction or cessation of airflow despite breathing efforts. Certain cancer treatments can affect the pharyngeal airway and contribute to the development of OSA. Radiation therapy to the head and neck can exacerbate UA narrowing through fibrosis, pharyngeal dilator dysfunction, and reduction in posterior genioglossus muscle tone [20–22]. Hormonal therapy may also increase risk for OSA. Female hormones (e.g. estrogen and progesterone) increase UA muscle tonicity and stimulate ventilation [23, 24]. Aromatase inhibitors are a class of anticancer drugs that block estrogen production, thereby reducing the availability of estrogen to protect against UA collapse during sleep [24].

Current screening tools for OSA do not incorporate cancer-specific features that can potentially distort or worsen UA anatomy architecture [21, 24]. While the STOP-Bang questionnaire [25] is a commonly used screening questionnaire validated for the preoperative setting and sleep clinics, its reliability in patients with cancer may be different than those in the general population [26, 27]. For instance, weight and age have variable prevalence in patients with cancer. Thus, the accuracy of the STOP-Bang questionnaire in the population with cancer is not only unknown but may be less relevant.

Identifying a screening tool and predictors for OSA in patients with cancer could be understood using *p*-values and Gaussian distributions. However, in this era of precision medicine, real-life populations often do not fit a linear structure. Machine learning (ML) algorithms enable analysis of heterogeneous conditions without imposing restrictions on the study population [28]. ML is highly effective in disease classification and determining predictors of disease [29]. The objectives of this study were to develop an accurate screening tool for OSA among patients with cancer using ML and to identify the most significant features predicting OSA in this population.

## Methods

### Study participants and design

This was a retrospective study of adult patients at Memorial Sloan Kettering Cancer Center who completed a home sleep apnea test (HSAT) from February 25, 2019 to February 15, 2021. All HSAT referrals were reviewed for appropriateness according to the American Academy of Sleep Medicine practice guidelines [30] by a board-certified sleep physician. This study was approved by the Memorial Sloan Kettering Cancer Center Institutional Review Board and complies with the Health Insurance Portability and Accountability Act.

All patients were asked to complete the STOP-Bang questionnaire (Supplementary eTable S1) [25] and Epworth Sleepiness Scale (Supplementary eTable S2) [31] on the date of HSAT administration. Inclusion criteria were as follows: (1) age ≥ 18 years, (2) history of cancer, (3) HSAT indicated, and (4) HSAT report available. Patients were excluded for (1) HSAT recording time < 4 hours, (2) suspicion of central sleep apnea upon review of HSAT, (3) external HSAT, and (4) incomplete STOP-Bang questionnaire.

Baseline demographics and clinical characteristics from the date of HSAT were electronically extracted from the institutional database. Types of cancer and comorbid conditions were obtained using international classification of diseases-9/international classification of diseases-10 codes. Chart review was performed by a physician to verify the type of cancer, history of radiation to the head/neck/thorax (RT-HNT), and aromatase inhibitor administration.

The Alice Night One (Phillips, USA) is a portable, type 3 sleep study device used for OSA diagnosis. A sleep technologist or sleep physician demonstrated how to use the device in person. Patients were subsequently issued the same HSAT device for overnight use in their homes.

All portable sleep studies were scored by a board-certified sleep physician using the Center for Medicare and Medicaid Services (CMS) scoring criteria [32]. The CMS scoring criteria were used because it is universally accepted by insurance payors. An apnea was defined as ≥90% reduction in airflow lasting > 10 seconds. A hypopnea was scored if there was a partial (≥30%) reduction in airflow for > 10 seconds accompanied by a 4% arterial oxygen desaturation from baseline. OSA severity was calculated using the respiratory event index (REI), defined as the total number of hypopneas and apneas scored divided by monitoring time (MT). MT was defined as total recording time minus periods of artifacts, as determined by lack of signal from plethysmography. Post-study questionnaires were completed by patients to record their perception of estimated total sleep time (TST) and compared with HSAT's estimated total MT. Patients were asked to repeat HSAT or pursue in-laboratory polysomnogram if the discrepancy between estimated TST and estimated total MT was greater than 1 hour. The REI correlates well with the apnea–hypopnea index (AHI) but may be lower since the denominator (i.e. MT) is larger than TST [33]. For simplicity and standard convention, “REI” was used as a surrogate for AHI and will be referred to as “AHI” in this paper.

### Statistical analysis

The chi-squared and Fisher’s exact tests were used to determine statistical significance of the categorical variables. The Shapiro–Wilk test was performed to check for the normality in data distribution. The ANOVA and Kruskal–Wallis tests were conducted to examine the statistical significance between the continuous variables and categorical variables. The Spearman correlation test was conducted on the continuous variables. A correlation heatmap was plotted to color code the correlation coefficients and examine the positive and negative correlation between the continuous variables in our dataset. The *p*-values and population proportion with a 95% confidence interval (CI) were computed to evaluate the statistical association between OSA and other variables. *p* < 0.05 was considered statistically significant.

### ML approach

ML has been utilized in medicine to predict two categories of clinical outcomes using binary classifiers [34]. [35], In this study, we conducted the following steps to predict “OSA” or “no OSA” in patients with cancer. Programming for statistical testing and ML was performed in Python (version 3.4, with scikit-learn package).

#### Assessing linearity of features.

Baseline demographics and clinical characteristics (Table 1) were used as features for analysis. The linearity of the features was tested using linear regression and visually plotted to confirm the presence of nonlinear relationships.

**Table 1.** Participant Characteristics of Dataset Used for Statistical Analysis, Training, and Validating the ML Model. *p*-values Represent Single Chi-squared Tests Examining for a Relationship With OSA. 95% Confidence Interval Pertains to the Number of People With OSA (Population Proportion) With Each Respective Participant Characteristic

Participant characteristics	All patients (*n* = 249)	Patients with OSA (*n* = 205)	Patients without OSA (*n* = 44)	*P*- Value	Population proportion 95% CI
Age, mean +/− SD, years (50+/− 23)					
28 to 40	18	11 (5.3%)	7 (15.9%)	<0.001	0.38 to 0.83
41 to 60	102	81 (39.5%)	21 (47.7%)	<0.001	0.71 to 0.87
61 to 93	129	113 (55.12%)	16 (36.36%)	<0.001	0.81 to 0.93
Gender				0.09	
Female	73	55 (26.82%)	18 (40.9%)		0.65 to 0.85
Male	176	150 (73.17%)	26 (59.09%)		0.79 to 0.90
Race				0.67	
Caucasian	171	137 (66.82%)	34 (77.27%)		0.74 to 0.86
Asian	21	18 (8.7%)	3 (6.8%)		0.70 to 1.00
African American	32	29 (14.14%)	3 (6.8%)		0.80 to 1.00
Other	13	11 (5.36%)	2 (4.5%)		0.65 to 1.00
Unreported	12	10 (4.8%)	2 (4.5%)		0.62 to 1.00
BMI, mean +/− SD, kg/m^2^ (31+/− 20)					
≥ 30	133	119 (58%)	14 (34%)	<0.001	0.75 to 0.92
< 30	116	88 (42.92%)	28 (63.63%)	<0.001	0.74 to 0.93
Neck size, mean +/− SD, cm (42 +/− 12)					
< 40	57	38 (21.46%)	19 (43.18%)	<0.001	0.61 to 0.85
≥ 40	146	131 (63.9%)	15 (34.09%)	<0.001	0.81 to 0.92
STOP-Bang characteristics					
Snoring	174	153 (74.63%)	21(47.72%)	0.001	0.83 to 0.92
Tiredness	186	155 (75.6%)	31 (70.45%)	0.60	0.77 to 0.88
Observed apnea	98	88 (42.92 %)	10 (22.72%)	0.024	0.83 to 0.95
Hypertension	120	108 (52%)	12 (27%)	0.003	0.84 to 0.95
BMI > 35	64	59 (28.7%)	5 (11.36%)	<0.001	0.85 to 0.98
Age > 50	193	168 (81.95%)	25 (56.81%)	<0.001	0.82 to 0.91
Neck size ≥ 40cm	193	167 (81.46%)	26 (59.09%)	<0.001	0.81 to 0.92
Gender—Male	176	150 (73.17%)	26 (59.09%)	<0.001	0.79 to 0.90
STOP-Bang score					
Low risk, 0–2	30	14 (6.8%)	16 (36.36%)	<0.001	0.28 to 0.64
Intermediate risk, 3–4	90	73 (35.6%)	17 (38.63%)	<0.001	0.73 to 0.89
High risk, 5–8	129	118 (57.5%)	11 (25%)	<0.001	0.86 to 0.96
Initial ESS, mean +/− SD (Mean ESS 8.3 +/− 4.7)					
0 to 10	182	152 (74.14%)	30 (68.18%)	<0.001	0.77 to 0.89
11 to 15	44	35 (17.07%)	9 (22.45%)	<0.001	0.66 to 0.92
16 to 23	23	18 (8.7%)	5 (11.36%)	<0.001	0.61 to 0.95
Comorbid conditions					
Asthma	27	23 (11.2%)	4 (9.09%)	0.94	0.71 to 0.98
Chronic kidney disease	24	20 (9.7%)	4 (9.09%)	0.88	0.25 to 0.65
COPD	22	21(10.24%)	1 (2.27%)	0.12	0.86 to 1.00
Diabetes	55	52 (25.36%)	3 (6.8%)	0.001	0.88 to 1.00
Hypertension	120	108 (52%)	12 (27%)	0.003	0.84 to 0.95
Cancer metastases	29	24 (11.7%)	5 (11.36%)	0.8	0.69 to 0.96
Type of cancer				0.53	
Head and neck	13	11 (5%)	2 (4%)		0.65 to 1.00
Lung	17	17 (8%)	0 (0%)		1.0 to 1.00
Prostate	65	57 (27%)	8 (18%)		0.79 to 0.95
Hematologic	40	34 (16%)	6 (13%)		0.73 to 0.96
Gynecological	8	7 (3.4%)	1 (2%)		0.59 to 1.00
Breast	40	29 (14%)	11 (25%)		0.58 to 0.86
Renal	17	12 (5.8%)	5 (11.3%)		0.48 to 0.92
Testicular	10	7 (3.4%)	3 (6.8%)		0.41 to 0.98
Bladder	12	10 (4.8%)	1 (2.27%)		0.62 to 1.00
Colon	8	7 (3.41%)	1 (2.3%)		0.51 to 0.98
Gastric	3	3 (1.46%)	0 (0%)		1.00 to 1.00
Skin	3	3 (1.46%)	0 (0%)		1.00 to 1.00
Rectal	3	2 (0.97%)	1 (2%)		0.13 to 1.00
Soft tissue	4	3 (1.46%)	1 (2%)		0.32 to 1.00
Other	3	3 (1.46%)	0 (0%)		1.00 to 1.00
Cancer-related therapy					
History of RT to head/neck/thorax	67	55 (26%)	12 (27.27%)	0.89	0.72 to 0.91
Aromatase inhibitors	24	21 (10%)	3 (6.8%)	0.67	0.74 to 1.00
Smoking history				0.14	
Never smoker	142	111 (54.14%)	31 (70.45%)	1.0	0.71 to 0.84
Ever smoker	84	72 (35.12%)	12 (27.27%)	0.42	0.78 to 0.93

#### Feature extraction using dimensionality reduction of variables.

In clinical datasets, multiple features and a high number of categorical variables can give rise to multicollinearity and the “curse of dimensionality” [36]; [37], when there are more features existing in higher dimensions, the amount of samples in each dimension decreases. Thus, more samples are required in each dimension for numerical analysis and ML. Statistical testing is challenging when evaluating sparse samples in higher dimensions.

In regression and classification experiments, feature selection and elimination is the first step [38]. Kernel principal component analysis (PCA), an advanced unsupervised ML method, was applied to extract the most significant features from the data and reduce the dimensions of the feature space into a non-linear subspace [39]. This technique eliminates insignificant features and determines the significant features (“principal components”) that explain the maximum variance with our target variable, OSA.

#### Classification of OSA.

Linear (logistic regression, LR) and non-linear (k-nearest neighbors, KNN; random forest, RF) ML classifiers were applied to the principal components following kernel PCA. The classifiers operated on the principal components to predict whether a patient is at risk of OSA.

#### Training and hyperparameter optimization.

The ML models chosen to perform best with hyperparameter optimization were trained on our dataset obtained from February 25, 2019 to February 15, 2021. We used 80% of the dataset to train the algorithms and the remaining 20% as the “validation set” based on standard convention [40]. The model hyperparameters were optimized using nested cross-validation technique (k-fold, *k* = 5) with grid search method to fine-tune model performance and estimate performance on five different folds. This technique prevents overfitting and biased outcomes by selecting the model with the best-performing parameters using a series of training, validation, and testing splits. The number of components and kernel type were optimized for PCA. The “linear” kernel was selected for the PCA + LR model; the “sigmoid” kernel was selected for the PCA + KNN and PCA + RF model. The optimum parameters were subsequently chosen based on the performance accuracy of OSA risk classification.

The diagnostic performance of the ML models and STOP-Bang questionnaire (as the only feature) were compared in the validation set. STOP-Bang analysis was performed using LR.

#### Prospective testing.

We tested our ML models on a prospective unseen test set of new patients from February 16, 2021 to June 30, 2021. Performance metrics of prediction accuracy and receiver operating characteristic-area under the curve (ROC-AUC) scores were obtained for each ML model and compared against the STOP-Bang questionnaire to evaluate the ability to screen for OSA.

## Results

### Patient population

We evaluated a total of 340 patients who completed a sleep study at our institution from Feb 25, 2019 to February 15, 2021; 249 participants fulfilled all inclusion and exclusion criteria (Supplementary eFigure S1). Table 1 summarizes the baseline characteristics for those found to have OSA (*n* = 205) and no OSA (*n* = 44) on HSAT in our training dataset. No statistical significance was found between the OSA group and the group without OSA in terms of variability of gender or race; 73% of the OSA group were male whereas 59% of the patients without OSA were male (Table 1).

### Statistical analysis

AHI was found to have the highest positive correlation (correlation coefficient 0.46) with the STOP-Bang score and a negative correlation with O2 nadir (Supplementary eFigure S2). Distinct positive correlations were between STOP-Bang score to neck size and BMI to neck size (Supplementary eFigure S2). There was no significant difference found between subgroups with BMI < 30kg/m^2^ and BMI ≥30 kg/m^2^ and Epworth Sleepiness Scale in terms of population proportion (95% CI) for risk of OSA (Table 1).

Patients with RT-HNT (82%, *p *> 0.05), reported snoring (87.9%, *p* = 0.001), age > 50 years (87%, *p* < 0.001), lung cancer (100%, *n* = 17), prostate cancer (87.69%, *n* = 65), neck size ≥40 cm (86.5%, *p* < 0.001), and hypertension (90%, *p* = 0.003) had OSA. Although not statistically significant, male gender, Asian and African American race, hypertension, COPD, asthma, and ever-smoker conferred an increased risk for OSA based on population proportion. Cancer-related patient characteristics associated with risk of OSA included lung cancer (95% CI: [1.0, 1.0]), prostate cancer (95% CI: [0.79, 0.95]), hematologic cancer (95% CI: [0.73, 0.96]), metastatic disease (95% CI: [0.69, 0.96]), and history of RT-HNT (95% CI: [0.72, 0.91]); in the breast cancer subgroup, prior use of aromatase inhibitors (95% CI: [0.74, 1.0]) also increased risk of OSA.

### ML approach

#### Non-linearity of data.

**Figure 1.**
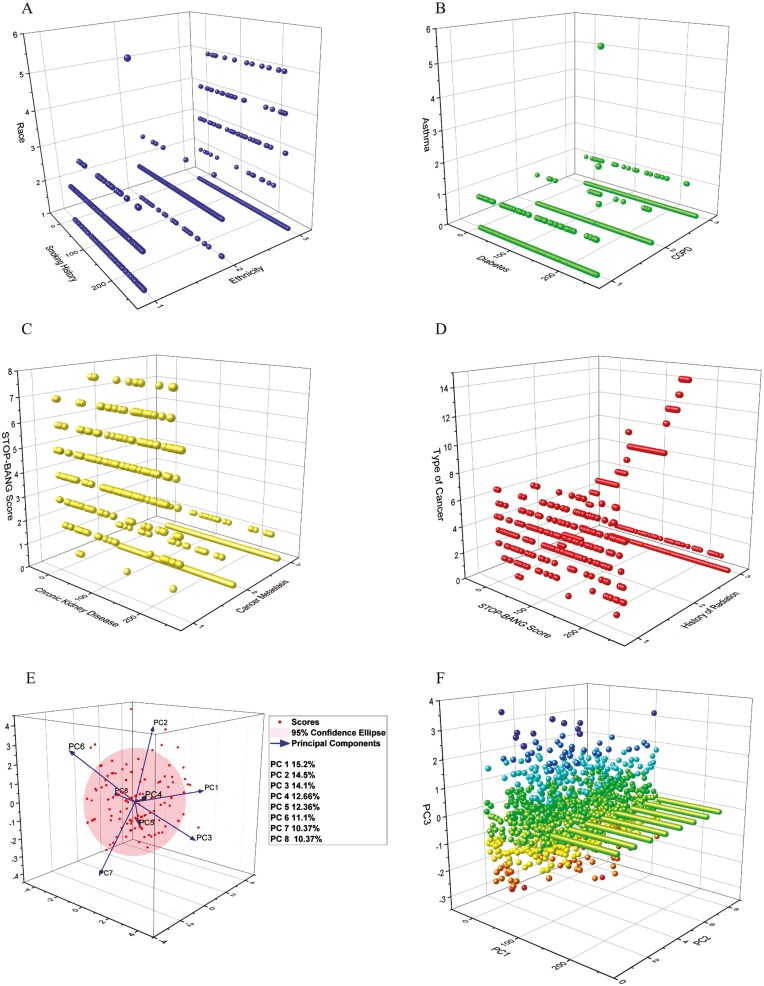
Non-linearity of data. Subplots A–D show the features scattered in 3D space indicating non-linear relationships. (E) is a projection of the eight principal components that contribute to the maximum variance (98%) of the features with the target variable “OSA.” (F) is a projection of the components on a non-linear subspace with reduced dimensionality that appears like clusters, upon which the ML classifiers were then applied for pattern recognition.

We calculated the coefficient of determination (R-squared) to estimate the goodness of fit of a linear model with the dataset. A negative R-squared value (−2.42) was obtained from the linearity test on the dataset using linear regression, which indicated that the linear model could not fit the data optimally and fit worse than a horizontal hyperplane [41]. Visual plotting confirmed the non-linear relationships between features (Figure 1A-D subplots).

#### Kernel principal component analysis.

Figure 3

**Figure 3.**
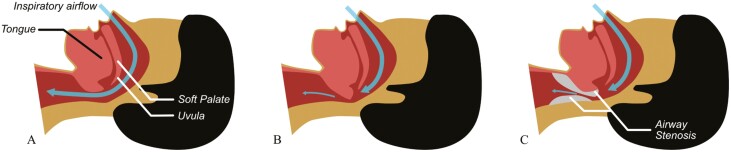
Airflow limitation in OSA. (A) In normal sleep, UA patency is maintained. (B) OSA occurs when there is a narrowing of the UA space with airflow limitation during sleep due to interactions between unfavorable anatomic UA susceptibility and sleep-related changes in UA function. (C) Patients who receive radiation therapy to the head and neck may have subsequent anatomic UA modification (e.g. stenosis), which can also cause airflow limitation during sleep and result in nascent or worsening existing OSA.

The participant characteristics in Table 1 were the features evaluated for the ML model. The application of kernel PCA extracted eight principal components through dimension reduction into the non-linear subspace (Figure 1E, F subplots). These components explain the maximum expected variance with OSA at 98%.

PCA analysis revealed that history of RT-HNT (0.66, PC5), ever-smoker (0.62, PC7), asthma (0.61, PC6), CKD (0.61, PC8), cancer metastases (0.58, PC5), STOP-Bang score (0.57, PC3), race (0.481, PC1), type of cancer (0.459, PC2), and diabetes (0.49, PC6) established heavy loading in the 8 principal components. The coefficients of eigenvectors in the brackets describe the maximum loading of the feature for the specific principal component.

#### Classification of OSA.

PCA followed by classification with non-linear ML classifiers outperformed LR with the ROC-AUC being the highest for PCA + RF algorithm (0.88), followed by PCA + LR (0.80) and PCA + KNN (0.82).

Non-linear ML classifiers, RF and KNN, consistently outperformed LR (with and without kernel PCA). The PCA + RF ML model had the best performance out of the four models for each performance metric in the validation set (Table 2). Sensitivity and specificity, respectively, were highest for PCA + RF (96.8%, 92.3%) compared to PCA + KNN (91.0%, 84.4%), and PCA + LR (92.8%, 66.7%). Positive and negative predictive values were highest for PCA + RF (94.0%, 92.0%), followed by PCA + KNN (92.6%, 91%), and PCA + LR (81.2%, 76.0%). The F1 score was highest for PCA + RF (0.93). The ML models with non-linear classifiers were also superior to the STOP-Bang questionnaire in screening for OSA based on all performance metrics (Table 2). All metrics were computed to 95% CI.

**Table 2.** Diagnostic Performance of ML Models and the STOP-Bang Questionnaire for Screening of Obstructive Sleep Apnea in Patients With Cancer From the Validation Set

Performance metrics	LR	PCA + LR	PCA + KNN	PCA + RF	STOP-BBang
Sensitivity, %	72.02 (71.0–73.65)	92.80 (90.3–94.0)	91 (90.80–93.30)	96.80 (94.50–98.10)	73.41 (72–74.02)
Specificity, %	76.10 (73.80–78.40)	66.70 (64–68.90)	84.4(82.01–87.20)	92.3 (91.03–94.09)	71.70 (68.90–72.70)
PPV, %	73 (71.03–75.80)	81.20 (79.03–82.2)	92.60 (91–94.30)	94 (92–96.77)	79 (77.02–81)
NPV, %	74.70 (72.90–77)	76 (73.98–78)	91 (89.56–93.40)	92 (90.77–94.60)	75.66 (74.86–77.50)
F1 Score	0.78 (0.76–0.81)	0.84 (0.83–0.86)	0.92 (0.9–0.94)	0.93 (0.91–0.95)	0.70 (0.66–0.80)
ROC-AUC Score	0.71 (0.69–0.73)	0.80 (0.79–0.82)	0.82 (0.80–0.84)	0.88 (0.86–0.90)	0.80 (0.78–0.82)

Varying sample sizes from the validation set were used to evaluate the performance of the ML classifiers using true positive rate versus false positive rate (Figure 4). LR without kernel PCA performed better for smaller sizes of test samples (ROC-AUC scores of 0.81 and 0.88 for 10 and 20 samples, respectively) only; the ROC-AUC for LR without kernel PCA declined when additional sample sizes were added (i.e. 0.77, 30 samples; 0.71, 50 samples). LR performance improved when combined with kernel PCA; ROC-AUC scores for 10, 20, 30, and 50 samples were 0.95, 0.96, 0.86, and 0.81, respectively.

#### Prospective unseen test set.

In assessing the performance of the ML models on our prospective unseen test set (*n* = 15), the PCA + RF model obtained the highest F1 score (0.82), sensitivity (81.7%), and specificity (81.3%) (Table 3). This was followed in performance by the PCA + KNN model, PCA + LR, and LR, respectively. The PCA + RF and PCA + KNN models outperformed the STOP-Bang questionnaire for all performance metrics (Table 3).

**Table 3.** Diagnostic Performance of ML Models and the STOP-Bang Questionnaire for Screening of Obstructive Sleep Apnea in Patients With Cancer From the Prospective Unseen Test Set

Performance metrics	LR	PCA + LR	PCA + KNN	PCA + RF	STOP-Bang
Sensitivity, %	60.01 (59.08–61.05)	80.80 (79.3–81.2)	81 (80.01–82.30)	81.7 (80–83.10)	60.8 (60–61.2)
Specificity, %	62 (61.80–63.40)	61.7 (60–63.1)	79.4(79.01–80.20)	81.3 (80.4–82.01)	68.54 (67.2–69.70)
PPV, %	64 (62.93–65.80)	81.20 (79.03–82.2)	82.60 (81.78–84.01)	83 (82–85.13)	66 (64.8–67.2)
NPV, %	64.6 (63.10–65)	65.7 (64.02 – 67.1)	81.96 (80.27–83.15)	82.86 (80.9–84.03)	64.52 (64.06–65.50)
F1 Score	0.61 (0.60–0.62)	0.78 (0.77–0.79)	0.81 (0.8–0.83)	0.82 (0.81–0.84)	0.61 (0.59–0.63)
ROC-AUC Score	0.60 (0.59–0.62)	0.70 (0.69–0.71)	0. 79(0.77.3–0.81)	0.80 (0.79–0.818)	0.71 (0.70–0.722)

## Discussion

This novel study utilized ML to develop a predictive screening tool for OSA in patients with cancer and identify predictors for OSA in this population. Characteristics unique to patients with cancer may contribute to OSA risk, including history of RT-HNT, type of cancer, and cancer metastases. Our ML screening tool incorporates these features to improve precision screening for OSA in patients with cancer. The PCA + RF ML model performed best at screening for OSA in patients with cancer compared to the STOP-Bang questionnaire, which is typically used for screening in the general population.

**Figure 2.**
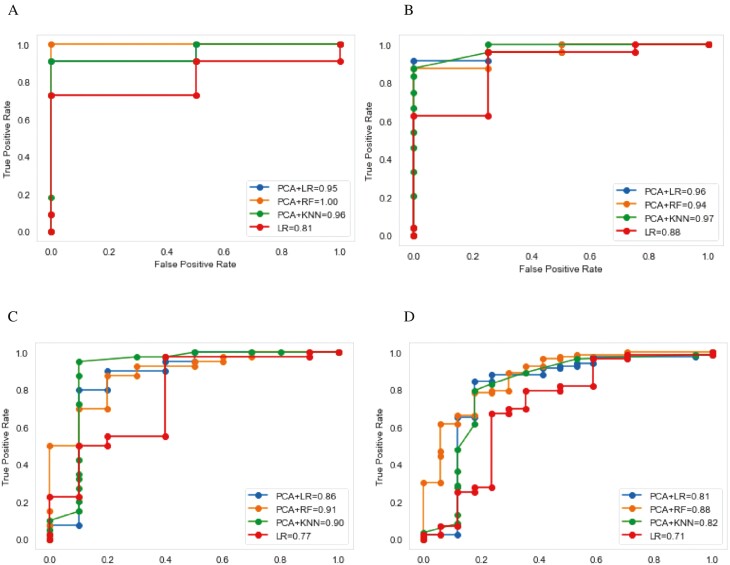
The receiver operating characteristic curves for ML models. The receiver operating characteristic curves for prediction of OSA using the different model techniques on test sets with (A) 10 samples, (B) 20 samples, (C) 30 samples, and (D) 50 samples. The legend displays the ROC-AUC score for each model in the respective subplots.

ML can facilitate improved clinical decision-making and precision medicine. Traditional statistical analyses operate on a best-fit model; however, real-life populations, especially cancer patients, are heterogeneous and non-linear. We were able to visualize evidence for this in our dataset (Figure 2, 2A–D subplots), which showed patient characteristics scattered in multiple dimensions. The different test samples (Figure 4) demonstrated that traditional LR sensitivity and specificity performance were improved with the addition of the unsupervised ML technique, PCA, for dimension reduction and feature extraction prior to classification. Consequently, we achieved a higher prediction accuracy for OSA using an ML model with PCA and non-linear classifiers compared to the linear classifier, LR.

Non-linear ML models perform better in real-life datasets because of no a priori statistical assumption of variables, an ability to train through continuous supervised or unsupervised learning mechanisms on intrinsic dimensions, and non-linear characteristics of variables, which leads to a reduction in biased outcomes [36]. The high sensitivity, specificity, positive-negative predictive value, F1, and ROC-AUC scores of our ML algorithms cross-validated on the validation set and tested on the prospective unseen test set further confirms the reliability of our PCA + RF model as a robust screening tool for screening OSA in patients with cancer.

### Predictors of OSA in patients with cancer.

Patient characteristics were used as input features to build our ML framework to screen for OSA in patients with cancer. The PCA + RF ML algorithm revealed the following features to be the most significant predictors of OSA: history of RT-HNT, ever-smoker, asthma, CKD, cancer metastases, STOP-Bang score, race, diabetes, and type of cancer. Although the *p*-values for history of RT-HNT, smoking history, CKD, race, and type of cancer failed to reject the null hypothesis in our statistical analysis, the heavy loading of these variables with individual principal components in our kernel PCA signifies a strong relationship between the interaction of these features with the principal components. This is insightful because the *p*-value criterion significance > 0.05 cannot always signify the actual impact of a feature when dealing with smaller sample sizes. Moreover, it is not a definitive marker of a feature’s “significance” or the probability of a null hypothesis being true, but rather how data are compatible with a null hypothesis.

### Cancer-related predictors of OSA.

We observed that history of RT-HNT, cancer metastases, and type of cancer prove significant in the prediction of OSA in patients with cancer. It is well-established that craniofacial anatomy plays a large role in the development and severity of OSA; restriction of the UA by altered skeletal morphology or excessive soft tissue endangers airway patency and predisposes to airway collapse [42]. Both early (i.e. edema) and sustained (i.e. deconditioned airway musculature, fibrosis, and stenosis) airway complications from RT-HNT are plausible mechanisms for increased susceptibility of OSA (Figure 5).

IH, one of the physiologic consequences of OSA, may enhance metastatic potential, as supported by animal model studies [43, 44]. IH increases the expression of hypoxia-vascular endothelial growth factor-A; subsequent angiogenesis can support tumor proliferation and metastasis [44]. IH also upregulates hypoxia-inducible factor- 1-α, activating the transcription of genes involved in angiogenesis, invasion, and metastasis [45].

The association between cancer type and OSA by our ML algorithm, namely lung, prostate, and hematologic cancers, is consistent with previous clinical studies [46, 47]. The increased incidence of hematologic malignancies with OSA was recently described in a South Korean population [48, 49]. One of the proposed theories for the relationship is the shared risk factor of obesity for OSA and hematologic malignancies [50].

### Limitations

Our study consisted of 249 patients with cancer, which is to the best of our knowledge, the largest sample size to be screened for OSA using ML in patients with cancer. This study, however, has limitations. First, there was a lower number of patients with certain cancer-relevant features such as history of RT-HNT and specific subtypes of cancer, which may affect model performance. To address this limitation, our study used the 95% CI of population proportion to verify the similar prevalence of features across subgroups. All cancer-relevant features were also chart reviewed to ensure integrity of the data. Our dataset had no missing values, which increases the strength of our ML algorithm and reduces inaccuracy. Furthermore, our ML model was tested on a prospective unseen test set which confirmed exceptional performance accuracy. Future studies should incorporate a database of a more uniform distribution of cancer-relevant features to enhance model performance.

Second, OSA was established using HSATs which can underestimate OSA severity and potentially mislabel patients with OSA as not having OSA. This can, in turn, affect our ML training. To address the inaccuracies in HSAT reporting, HSAT post-study questionnaires with estimated time slept were corroborated with total recording time to improve HSAT reliability. We also used a binary classification of OSA (with or without) to train our screening model. Subsequent studies aiming to predict OSA severity can use larger sample sizes, including samples across all severity groups, and integration of polysomnogram data to train ML classifiers.

Third, we did not validate our ML model with an external test set. Although we used a prospective unseen test set for validation, external validation sets from different institutions would be warranted in future studies to ensure a similar discriminative ability of our model.

## Conclusions

This is the first time to the best of our knowledge that an ML algorithm has been designed to screen for OSA in patients with cancer. ML identified features unique to patients with cancer, which are currently not included in traditional screening tools for OSA. Our ML algorithm was based on 249 patients, devoid of null values, and reproducible in performance on unseen data. It incorporated features related to cancer and patient demographics (e.g. race) to increase precision for this population and reduce the probability of biased outcomes. Given the potential impact of OSA on morbidity and mortality in patients with cancer, it is important to screen for OSA in this group. Future direction will focus on expanding the sample size. If the ML algorithm performance remains robust, this ML model could serve as an efficient and cost-effective tool to accurately screen for OSA and improve access to care. Integration of this ML algorithm into an electronic health record system may facilitate screening of OSA and reduce health inequities.

## Supplementary Material

zpad042_suppl_Supplementary_Tables_1-2_Figures_1-2

## Data Availability

Available upon reasonable request.
