# Supplementary material for: Screening for obstructive sleep apnea in patients with cancer — a machine learning approach
Source: Sleep Adv. 2023 Oct 31;4(1):zpad042. doi: 10.1093/sleepadvances/zpad042 (PMC10735319; doi:10.1093/sleepadvances/zpad042)
Supplement: zpad042_suppl_Supplementary_Tables_1-2_Figures_1-2 [file zpad042_suppl_supplementary_tables_1-2_figures_1-2.docx]

**ONLINE DATA SUPPLEMENT**

***Screening for Obstructive Sleep Apnea in Patients with Cancer – a Machine Learning Approach***

*Karen A. Wong, MD^1^, *Ankita Paul, BS^2^, Paige Fuentes, MS^3^, Diane C. Lim, MD^4,5^, Anup Das, PhD^2^, Miranda Tan, DO^1^

^1^Pulmonary Service, Department of Medicine, Memorial Sloan Kettering Cancer Center, New York, NY, USA; ^2^Department of Electrical and Computer Engineering, Drexel University, Philadelphia, PA, USA; ^3^Department of Medicine, Memorial Sloan Kettering Cancer Center, New York, NY, USA; ^4^Department of Medicine, Miami Veterans Affairs Healthcare System, Miami, FL, USA; ^5^Department of Medicine, University of Miami, Miami, FL, USA

**Corresponding Author:** Miranda Tan, D.O.

**Present Address:** Stanford University School of Medicine, Palo Alto, CA, USA; Tel: (650)-724-6962, Fax: (650)-721-7570, Email: [tanm@stanford.edu](mailto:tanm@stanford.edu)

**SUPPLEMENTARY METHODS**

The STOP-BANG questionnaire (eTable 1) is a validated 8-point survey that screens for OSA. A total score <3 confers a low risk of OSA. Scores of 3-4 and 5-8 indicate an intermediate and high risk of OSA, respectively.^1^ The Epworth sleepiness scale (ESS) (eTable 2)provides a subjective estimate of sleepiness by chance of dozing off or falling asleep in 8 different activities. Each situation is rated on a 4-point Likert scale of 0-3, with 3 representing a high chance of falling asleep. ESS values >10 indicate daytime sleepiness. This instrument has been used to measure sleepiness in various populations.^2^

***Sleep study setup and scoring*.**

The Alice Night One (Phillips, USA) is a portable, type 3 sleep study device used for diagnosis of OSA. It is comprised of four channels to measure heart rate, oxygen saturation (pulse oximetry), airflow (via a nasal cannula pressure transducer), and thoracic respiratory effort (via a chest band with pneumatic sensor).

An AHI <5 events/hour indicated a normal sleep study; AHI >5 events/hour was defined as OSA. Values from 5-14, 15-29, and >30 events/hour classified mild, moderate, and severe OSA, respectively.^3^

**SUPPLEMENTARY FIGURES**


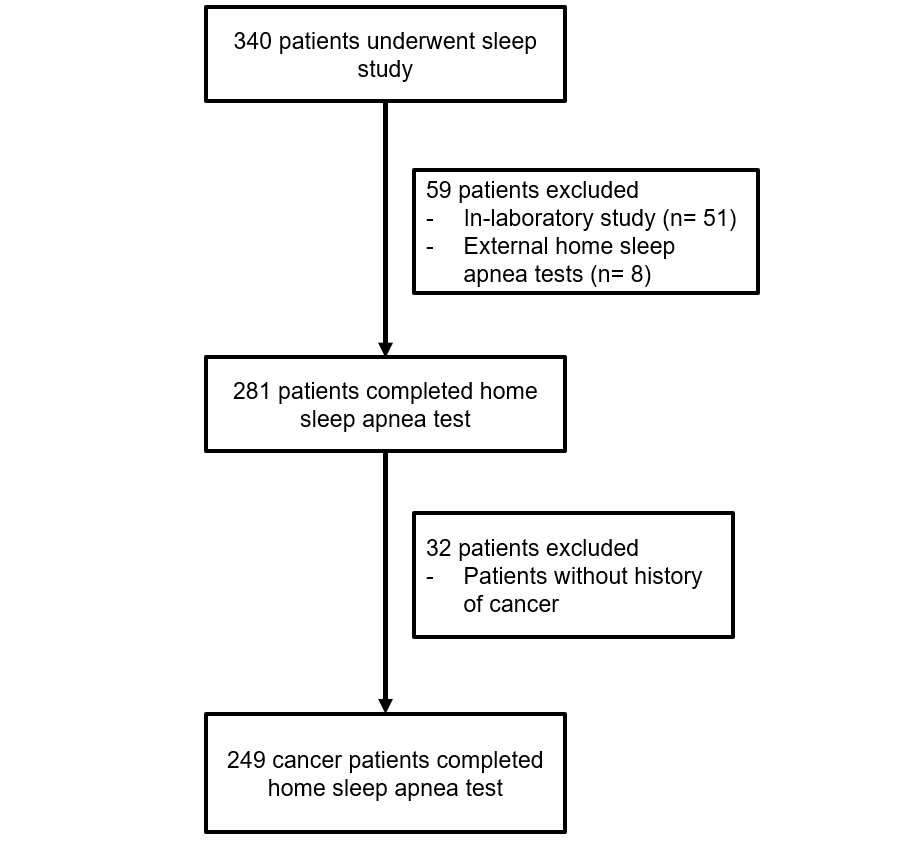


**eFigure 1. Flow diagram of final participants included in analysis and the machine learning training model.**


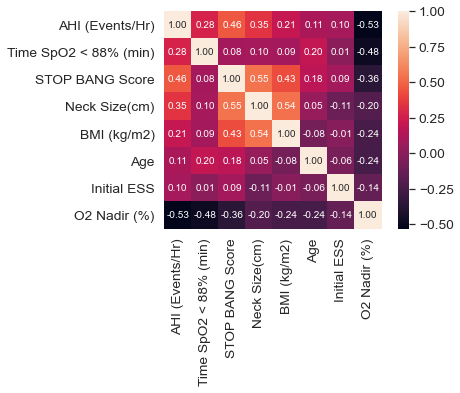


**eFigure 2. Heatmap correlating apnea-hypopnea index (AHI) with continuous variables using spearman correlation test.**


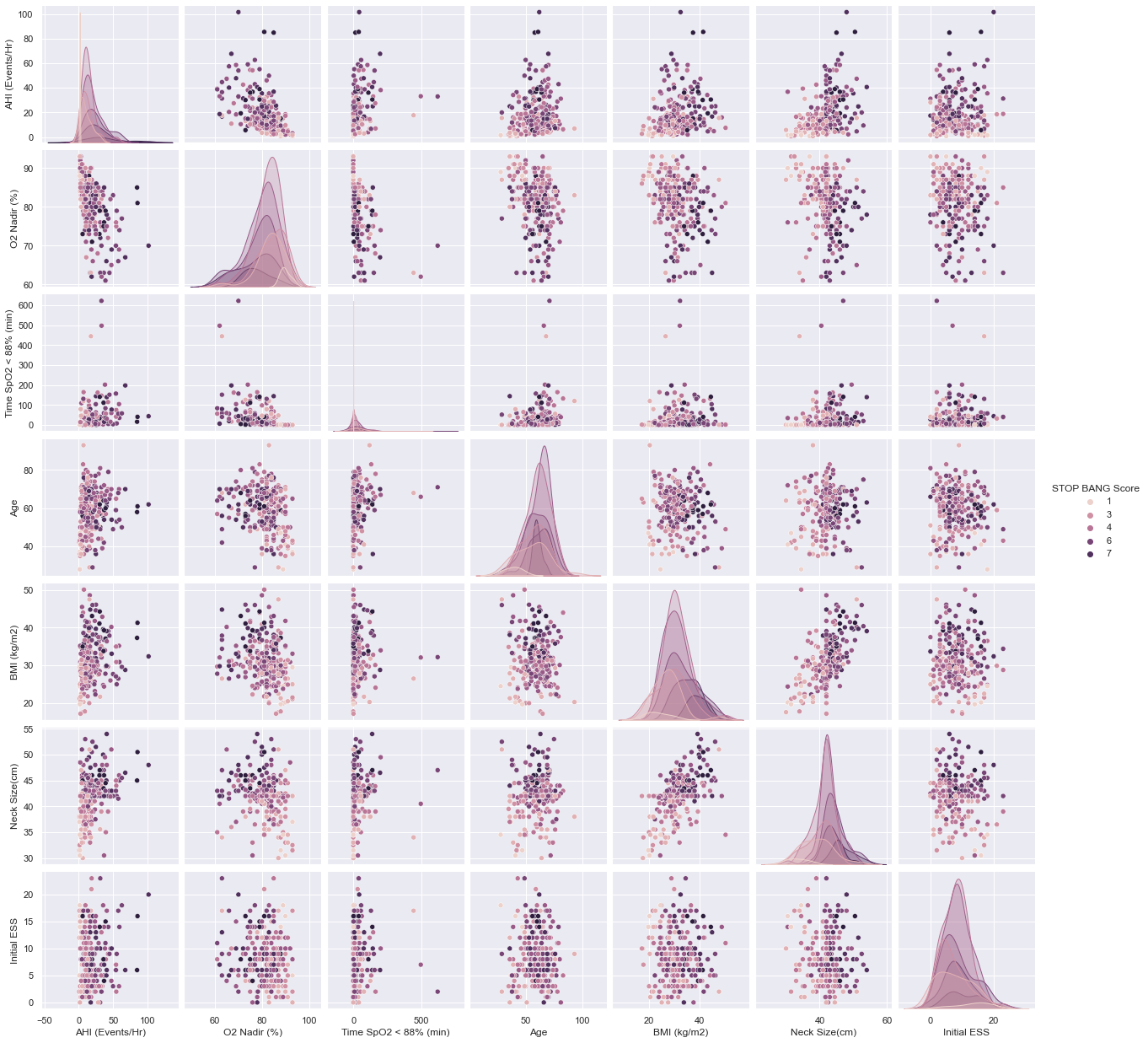


***eFigure 3. The relationship between the apnea-hypopnea index (AHI) and other continuous variables.***

|  | **Yes** | **No** |
| --- | --- | --- |
| **Snoring?**  Do you snore loudly, enough to be heard through closed doors? | 0 | +1 |
| **Tired?**  Do you often fee tired, fatigued, or sleepy during the day? | 0 | +1 |
| **Observed apneas?**  Has anyone observed you stop breathing during sleep? | 0 | +1 |
| **Pressure treatment?**  Do you have (or are you being treated for) high blood pressure? | 0 | +1 |
| **BMI?**  BMI >35kg/m^2^? | 0 | +1 |
| **Age?**  Age >50? | 0 | +1 |
| **Neck circumference?**  Circumference >40cm? | 0 | +1 |
| **Gender?**  Male gender? | 0 | +1 |

***eTable 1:*** ***STOP-Bang questionnaire.***

|  | **Chance of dozing** | | | |
| --- | --- | --- | --- | --- |
| How likely you are to doze or fall asleep in the following situations? | 0 | 1 | 2 | 3 |
| Sitting and reading | 0 | 1 | 2 | 3 |
| Watching television | 0 | 1 | 2 | 3 |
| Sitting inactive in a public place (eg, a theater or a meeting) | 0 | 1 | 2 | 3 |
| As a passenger in a car for an hour without a break | 0 | 1 | 2 | 3 |
| Lying down to rest in the afternoon when circumstances permit | 0 | 1 | 2 | 3 |
| Sitting and talking to someone | 0 | 1 | 2 | 3 |
| Sitting quietly after a lunch without alcohol | 0 | 1 | 2 | 3 |
| In a car, while stopped for a few minutes in traffic | 0 | 1 | 2 | 3 |

***eTable 2:*** ***Epworth Sleepiness Scale (ESS).***

**SUPPLEMENTARY RESULTS**

| **Variables** | **Sets** | | **P - Value** |
| --- | --- | --- | --- |
|  | Training | Testing |  |
| **RT-HNT** | 67 | 5 | 0.7 |
| **Asthma** | 27 | 3 | 0.8 |
| **Chronic kidney disease** | 24 | 3 | 0.04 |
| **Diabetes** | 55 | 5 | 0.07 |
| **Smoking History** |  |  | 0.005 |
| Never smoker | 142 | 19 |  |
| Ever smoker | 84 | 8 |  |
| **STOP-Bang Score** |  |  | 0.001 |
| Low risk, 0-2 | 30 | 3 |  |
| Intermediate risk, 3-4 | 90 | 13 |  |
| High risk, 5-8 | 129 | 18 |  |
| **Cancer Metastases** | 30 | 4 | 0.6 |
| **Type of Cancer** |  | | 0.001 |
| Head and neck | 13 | 1 |  |
| Lung | 17 | 0 |  |
| Prostate | 65 | 13 |  |
| Hematologic | 40 | 5 |  |
| Gynecological | 8 | 3 |  |
| Breast | 40 | 6 |  |
| Renal | 17 | 2 |  |
| Testicular | 10 | 2 |  |
| Bladder | 12 | 1 |  |
| Colon | 8 | 0 |  |
| Gastric | 3 | 0 |  |
| Skin | 3 | 1 |  |
| Rectal | 3 |  |  |
| Soft Tissue | 4 | 0 |  |
| Other | 3 | 0 |  |
| **Race** |  |  | 0.06 |
| White | 171 | 24 |  |
| Asian | 21 | 2 |  |
| Black | 32 | 1 |  |
| Other | 13 | 4 |  |
| Unreported | 12 | 3 |  |

***eTable 3****.* ***Demographic characteristics of participants in the training and testing datasets used for machine learning models.***

| **Participant Characteristics** | **All Patients (n=249)** | **Patients with OSA (n=205)** | **Patients without OSA (n=44)** | ***P*- Value** | **Population Proportion 95% Confidence Interval (CI)** |
| --- | --- | --- | --- | --- | --- |
| **AHI, mean +/- SD, events/h**  **18.49 +/- 15.5** |  | | | | |
| 0-5 | 44 | 0 (0%) | 44 (100%) | <0.001 | 0.95-1.00 |
| 5-14 | 91 | 91 (44.39%) | 0 (0%) | <0.001 | 0.9-1.00 |
| 15-29 | 70 | 70 (34.15%) | 0 (0%) | <0.001 | 0.9-1.00 |
| > 30 | 44 | 44 (21.46%) | 0 (0%) | <0.001 | 0.89-0.97 |

**eTable 4. Sleep study characteristics of dataset used for statistical analysis, machine learning training and cross-validation.**

**References:**

1. Chung F, Yegneswaran B, Liao P, et al. STOP Questionnaire: A Tool to Screen Patients for Obstructive Sleep Apnea. *Anesthesiology*. 2008;108(5):812-821. doi:10.1097/ALN.0b013e31816d83e4

2. Johns MW. Reliability and factor analysis of the Epworth Sleepiness Scale. *Sleep*. 1992;15(4):376-381.

3. Sleep-related breathing disorders in adults: recommendations for syndrome definition and measurement techniques in clinical research. The Report of an American Academy of Sleep Medicine Task Force. *Sleep*. Aug 1 1999;22(5):667-89.
